# Surgical site infection prevention practice and associated factors among nurses working at public hospitals of the western part of southern nation, nationalities, and peoples’ region, Ethiopia: A cross-sectional study

**DOI:** 10.3389/fsurg.2022.1013726

**Published:** 2022-11-14

**Authors:** Tamene Tesfaye, Merga Dheresa, Teshager Worku, Deribe Bekele Dechasa, Henock Asfaw, Abera Jambo Bune

**Affiliations:** ^1^ Department of Nursing, Mizan Tepi University Teaching Hospital, Mizan Tepi, Ethiopia; ^2^ School of Nursing and Midwifery, College of Health and Medical Science, Haramaya University, Dire Dawa, Ethiopia; ^3^ Clinical Pharmacy Department, School of Pharmacy, College of Health and Medical Sciences, Haramaya University, Harar, Ethiopia

**Keywords:** nurse, post-operative, surgical site infection, wound infection, prevention practice

## Abstract

**Background:**

Surgical site infection is a major hazard for surgical patients and compromises their quality of life. Its effect is higher in developing countries compared to developed countries. Most of the studies done in Ethiopia regarding surgical site infection prevention practice on nurses who were not directly exposed to wound care, thus it produces less reliable results. Therefore, we aimed to assess surgical wound infection prevention practice among nurses who are directly involved in the care.

**Objective:**

To assess surgical site infection prevention practice and associated factors among nurses working at public hospitals in the western part of the southern nation, nationalities, and peoples’ regions from March 1–31, 2020.

**Methods:**

An institutional-based cross-sectional study design was conducted from March 1–31, 2020 among randomly selected 402 study participants. A structured and pretested questionnaire was used. EpiData Version 3.1 and Statistical Package for Social Science Version 20 were used for analysis. Bivariable and multivariable analysis was undertaken and *p*-value less than 0.05 at a 95% confidence interval was considered statistically significant.

**Results:**

The overall good self-reported surgical site infection prevention practice of nurses was 46% (95% CI: 41.3, 50.7). Nurses who were BSc degree (AOR = 2.04; 95% CI: 1.31, 3.18), working in the units having surgical site infection prevention guidelines (AOR = 2.45; 95% CI: 1.34, 4.47), had ever taken infection prevention training (AOR = 2.23; 95% CI: 1.42, 3.49), had good knowledge (AOR = 1.82;95% CI: 1.13, 2.90) and had good attitude (AOR = 2.61;95% CI: 1.67, 4.10) performed good surgical site infection prevention activities as compared to their counterparts.

**Conclusion:**

Nurses’ surgical site infection prevention practice was found to be low. To upgrade nurses’ practice the hospitals should develop their surgical site infection prevention guidelines based on WHO recommendations and provide training on it.

## Introduction

Surgical site infections (SSIs) are infections that occur within the first 30 days after a surgical procedure if no implant is placed or within 1 year after a surgical procedure if an implant is placed (1). It is classified as superficial incisional SSIs which involve only the skin or subcutaneous tissue, deep incisional SSIs which involve fascia or muscular layer, and organ space SSIs which involve any part of the body manipulated during a procedure, excluding the previously mentioned layers (2).

Surgical site infection is a major hazard for surgical patients. The major consequences of SSIs are readmission, prolonged hospital stay, increased cost of treatment, long-term disability, emotional stress for patients and their families, high cost for the health care system, and a considerable economic burden for the hospitals (3, 4). Patients with SSIs have a 2–11 times greater risk of death compared with surgical patients without SSIs and 77% of deaths in patients with SSIs are directly attributable to SSIs (5).

Nurses can play a significant role in SSIs prevention by using different strategies including double gloving, wearing latex-free gloves, using alcohol-based hand sanitizer before donning non-sterile gloves, and maintaining hand hygiene before and after contacting patients (6). A variety of studies done across the world revealed that the level of surgical site infection prevention among nurses was different from global to local and continent to continent (7). A cross-sectional study conducted in Brazil revealed a high SSIs prevention practice. According to this study, the prophylactic antibiotic was administered up to 60 min before surgical incision in 90.3% of the cases and 91% of patients had also taken preoperative showering on the day of their surgery (8). Similarly, a cross-sectional study conducted in Palestine and India exhibited that 91.1% and 64.51% of nurses had a high level of SSIs prevention practice respectively (9, 10). A cross-sectional study conducted among 131 nurses in Pakistan revealed that only 28.2% of nurses had always practiced preoperative prophylactic antibiotics within 1 h before surgical operation while 71.8% of them did not practice (11).

Studies from Bangladesh revealed that only 44.5% of nurses were always practicing SSIs prevention activities (12). Evidence from Tanzania also exhibited that about 57.7% of nurses have poor SSI prevention practices (13). Another cross-sectional survey conducted in north-central Nigeria among 250 post-operative nurses in a tertiary health institution also revealed a relatively high SSIs prevention practice since only 15.1% of the respondents said they occasionally did not wash their obviously soiled hands (14).

Evidence of nurses’ SSIs prevention practice from Ethiopia indicated that more than half of the nurses had a poor practice of SSIs prevention. Two cross-sectional studies purposively selected 418 nurses in 2017 and randomly selected 515 nurses in 2019 conducted in hospitals of Harar city and Dire Dawa city administration revealed that the majority (71.3% and 59.2%) of the study participants had poor SSIs prevention practices respectively (15). A cross-sectional study conducted in Gondar university referral hospital among randomly selected 423 nurses revealed that the proportion of nurses who are practicing good SSI prevention strategies was 48.7% (16). Similarly, a cross-sectional study with systematic random sampling conducted among 208 nurses in Bahir-dar city hospitals revealed that 45.1% of study participants had a good practice (17).

Ethiopian Federal Ministry of Health May 2016, also launched a nationwide program of saving lives through safe surgery to address the national burden of SSIs by promoting improvements in surgical delivery and safety (18). Nurses, working nearest to the patient care area are in a vital position to play a leading role in taking initiatives that aimed to ensure patient safety including the prevention of SSIs (19, 20). Nurses’ surgical site infection prevention practice is significantly affected by personal factors such as nurses’ knowledge and attitude toward WHO-recommended SSIs prevention activities (14). Insufficient on-job training and inadequate resource to implement surgical safety checklists, and insufficient orientation programs during unit rotation are institutional-related factors that can affect the nurses’ SSIs prevention practice (21). These factors may vary from site to site. In Ethiopia, surgical site infections are currently recognized as one of the key performance indicators of inpatient quality of care, and SSIs surveillance is an integrated part of the quality improvement program (22).

Observation of nurses’ actual SSIs prevention practice is strongly recommended by previous similar studies but the statistically significant difference between self-reported and actual SSIs prevention practice did not identify. Therefore, this study will address this gap by observing 15% of study participants and determining SSIs prevention practice and associated factors among nurses working at selected units of public hospitals in the western part of the Southern Nation, Nationalities, and Peoples Region (SNNPR).

## Materials and methods

### Study area and period

The study was conducted in public hospitals in the western part of the Southern Nation, Nationalities, and Peoples’ Region (SNNPR) from March 01 to 31, 2020. This study area includes four zones Sheka zone, Bench-Sheko zone, Kaffa zone, and Debub mihrab omo zone. The study was conducted in five public hospitals. In all the five hospitals, there are 566 nurses; Mizan-Tepi University Teaching Hospital (MTUTH) has 198 nurses, Tepi General Hospital (TGH) has 117 nurses, Gebrestadik Shawo General Hospital (GSGH) has 133 nurses, Bachuma Primary Hospital (BPH) has 53 and Wacha Meleszenaw Primary Hospital (WMPH) has 65 nurses. The data was collected from March 01–31, 2020.

### Study design and population

An Institutional based cross-sectional study design was used. The study population were all nurses who were working in public hospitals in the western part of Southern Nation, Nationalities, and Peoples’ Region (SNNPR) and who were working in selected units such as Operation room, surgical ward, Intensive care unit, Pediatric ward, Gynecology/obstetrics ward, Surgical referral clinic (SRC), and Emergency units were included in the study. Those staff nurses who were ahead nurses, who were not voluntary to participate in the study, and who were not available during the study period due to training, sick leave, annual leave, and maternal leave during the study period were excluded.

### Sample size determination and sampling procedure

For the first objective sample size was calculated based on a formula for estimation of a single population proportion by considering: a 95% confidence level, 5% margin of error, and 45.1% prevalence (17). By using a single population proportion formula: $n=\;(({Z_{a}}_{/2}{)}^{2}p\left(1-p\right))/d^{2}$.

Then by adjusting the result for a non-response rate of 10% it will be (380*10)/100 = 38. By adding 380 and 38 it results in 418 samples. For the second objective sample size was calculated by Epi-info version 7 software by considering a 95% confidence interval, 80% power, and 1:1 ratio of unexposed to exposed groups for each factor. The sample size calculated for the first objective was taken since it is larger than that of the second objective. So, the final sample size for the study was 418. All public hospitals were included in the study and the determined sample size was proportionately allocated to each public hospital based on their total number of the nurse.

### Data collection tool and procedure

The data collection instrument was prepared in English version by reviewing previous comparable pieces of literature (21). The tool has five sections; Section one was about socio-demographic characteristics of nurses, institutional related conditions, and nurses’ knowledge about SSIs prevention practices which was adopted and revised from previous similar studies (16, 21). Section two was about nurses’ attitude towards the SSIs prevention strategies which was adopted and revised from previous similar studies (14). Section three was about the nurses’ self-reported practices about SSIs prevention which was adopted and revised from previous similar studies (16, 17). The outcome variable measuring questionnaires were validated for internal consistency among each item of the questions with a Cronbach's alpha (*α* = 0.73) by previous similar studies (3). Section four was an observational checklist to assess institutional related factors which were adopted and revised from the previous similar studies (10). Section five was an observational checklist that is designed to measure the actual nurses’ SSIs prevention practices which were developed by referring to different studies and international guidelines (21, 23, 24). An observational checklist and a self-administered questionnaire were used for data collection procedures.

### Data processing and analysis

The collected data were coded, entered, and cleaned by using EpiData statistical software Version 3.1 and then exported into SPSS version 20 for analysis. Descriptive statistical analysis was used to describe the characteristics of participants. Bivariable logistic regression analysis was run to determine the association between the independent variables and outcome variables. The *p*-value and an odds ratio with 95% CI were computed. Then the variables with a *p*-value ≤ 0.25 were taken into the multivariable model to control for all possible confounders. The multi-co-linearity test was carried out to see the correlation between independent variables by using tolerance and variance inflation factors (VIF). However, there was no independent variable with a tolerance value of less than 0.1 or VIF of greater than 10. For model fitness, Hosmer-Lemeshow's model of fitness was checked which was 0.411 indicating the test fit the model.

The level of statistical significance was declared at a *p*-value < 0.05. Finally, by using the coefficient of kappa and its interpretation, the agreement between an observational and self-reported nurses’ SSI prevention practice was determined (*k* = 0.424) indicating moderate agreement.

### Data quality control

The data was pretested on 5% of the study population at Tercha general hospital which is a minimum of 80 km far from the study sites. Data was collected by extensively trained data collectors and daily collected data was also checked for completeness and validity by supervisors and principal investigators. The collected data were coded and entered in EpiData Software Version 3.1. Double data entry to EpiData software by two independent data clerks was done. Then the two data sets were validated in the software for consistency and mismatches. Finally, to ensure the validity of data from self-reported practice, the statistically significant difference between nurses’ self-reported practice and actual practice was determined.

### Ethical consideration

Ethical approval was obtained from the Institutional Health Research Ethics Review Committee (IHRERC) of the College of Health and Medical Sciences, Haramaya University. The aim of the study, the potential risk, and the benefit of the study were clearly explained to the participants and informed voluntary written and signed consent was obtained from study participants. The participants were informed of their right to refuse participation in the study at any time.

## Results

### Socio-democratic characteristics of study participants

A total of 402 nurses returned a complete questionnaire, giving a response rate of 96.2%. Of this total number of respondents, 229 (57%) were females. The mean (±SD) age of study participants was 28.7 (±4.36) years old. Almost half, 197 (49%) of study participants were BSc degree holders and about three fourth of the participants 305 (75.9%) had work experience <5 years. Regarding infection prevention (IP) training only 159 (39.6%) study participants had ever taken IP training (Table 1).

**Table 1:** Socio-demographic characteristics of nurses working at public hospitals in the western part of SNNPR, Ethiopia 2020, (*n* = 402).

Variables	Category	Frequency (%)
Age (*n* = 402)	<30 years	262 (65.2)
	≥30 years	140 (34.8)
Sex	Male	173 (43)
	Female	229 (57)
Educational level	Diploma	205 (51)
	Degree	197 (49)
Work experience	<5 years	305 (75.9)
	≥5 years	97 (24.1)
Type of current working hospital	Primary hospital	74 (18.4)
	General Hospital	178 (44.3)
	Teaching hospital	150 (37.3)
IP training	Not trained	243 (60.4)
	Trained	159 (39.6)

### Institution related factors

In this study, 281 (69.9%) participants reported that surgical supply is available in their working units. However, only 65 (16.2%) participants reported that there is SSI prevention guideline in their working units. About 80% of nurses who reported the availability of guidelines in their units reported that they were using the guideline to update their knowledge.

### Personal factors

**Figure 1:**
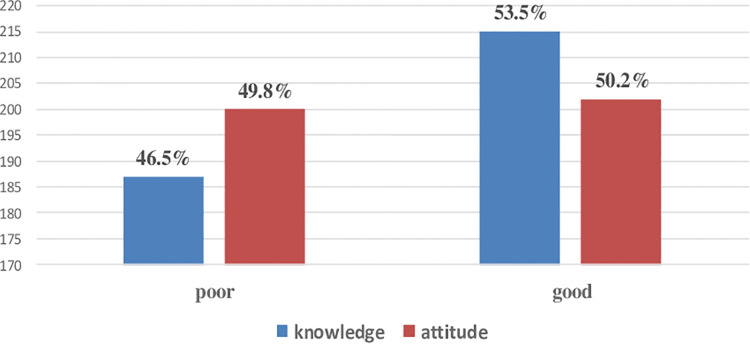
Knowledge and attitude status of nurses working at public hospitals of among nurses working at public hospitals in the western part of SNNPR, Ethiopia 2020, (*n* = 402).

Regarding professionals’ related factors, about 215 (53.5%) and 202 (50.2%) participants had good knowledge and good attitude toward SSI prevention strategies respectively. The mean (±SD) knowledge and attitude scores were 6.52 (±1.56) and 6.79 (±2.05) respectively (Figure 1).

### Overall self-reported SSI prevention practice status

**Figure 2:**
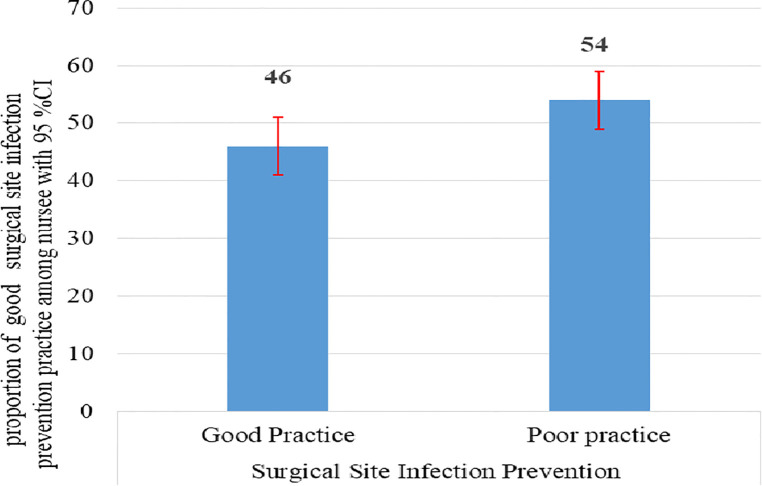
Overall self-reported surgical site prevention practice status among nurses working at public hospitals in the western part of SNNPR, Ethiopia 2020, (*n* = 402).

The mean (±SD) nurses’ self-reported SSIs prevention practice score was 9.33 (±2.775). According to this study, the overall good self-reported SSI prevention practice of nurses who were working at public hospitals in this study area was 46% (95% CI: 41, 50.7) (Figure 2).

### Surgical site infection prevention practice status by observation

To check the reliability of nurses’ self-reported SSIs prevention practice, 15% of study participants were observed and the concordance between concurrent studies (both reported an observational study) was determined. According to observational checklist findings, good nurses’ actual SSIs prevention practice was 41.3% (95% CI: 28.6, 52.4). Their corresponding good self-reported SSIs prevention practice was 46% (95% CI: 34.9, 60.3). Even though the overall findings of these two concurrent studies are consistent, it does not mean they are symmetrical. Therefore, the concordance between these two measurements was determined by computing the measure of agreement (kappa) which was 0.424 (significant at *p* = 0.001) (Table 2).

**Table 2:** Self-reported and actual surgical site infection prevention practice status cross-tabulation and symmetric measures among nurses working at public hospitals in the western part of SNNPR, Ethiopia 2020, (*n* = 402).

Nurses’ self-reported practice status * actual practice status cross-tabulation (*n* = 63)				
		Actual practice status		
		Poor practice	Good practice	Total (%)
Self-reported practice status	Poor practice	26	7	33 (52.4)
	Good practice	11	19	30 (47.6)
	Total (%)	37 (58.7)	26 (41.3)	63 (100)

### A measure of agreement between nurses’ self-reported and actual SSIs prevention practice

If K < 0 ⇒ Less than chance agreement, 0–0.2 ⇒ Slight agreement, 0.2–0.4 ⇒ Fair agreement, 0.4–0.6 ⇒ Moderate agreement, 0.6–0.8 ⇒ Substantial agreement, and 0.8–1.0 ⇒ Almost perfect agreement (25). Therefore, a kappa of 0.424 indicates that the measurements have a moderate agreement. Since there is a moderate agreement at *p* = 0.001, the agreement is not by chance (Table 3).

**Table 3:** Measure of agreement between self-reported and actual surgical site infection prevention practice among nurses working at public hospitals in the western part of SNNPR, Ethiopia 2020, (*n* = 402).

Symmetric measures					
		Value	Asymptotic standardize error^a^	Approximate T^b^	Approximate significance
Measure of agreement	Kappa	0.424	0.114	3.392	0.001
# of Valid cases		63			
					

^a^ Not assuming the null hypothesis.

^b^ Using the asymptotic standard error assuming the null hypothesis.

### Nurses’ actual and their self-reported practice status on each item of practice questions

The overall nurse's actual SSI good and poor prevention practice statuses were 26 (41.3%) and 37 (58.7%) respectively (Table 4).

**Table 4:** Actual and self-reported surgical site infection prevention practice status among nurses working at public hospitals in the western part of SNNPR, Ethiopia 2020, (*n* = 402).

Practice questions (*n* = 63)	Category	Actual practice status		Category	Self-reported practice status	
		Poor	Good		Poor	Good
		Count	Count		Count	Count
Wash his/her hand just before wearing a glove?	No	36	23	Less practiced	27	13
	Yes	1	3	Well practiced	6	17
Wash his/her hand after changing wound dressing?	No	9	4	Less practiced	17	3
	Yes	28	22	Well practiced	16	27
Assess and monitor surgical wound condition.	No	32	6	Less practiced	18	2
	Yes	5	20	Well practiced	15	28
Use sterile forceps and other dressing materials for dressing surgical wounds.	No	0	0	Less practiced	5	1
	Yes	37	26	Well practiced	28	29
Use aseptic techniques during surgical wound dressing?	No	1	0	Less practiced	6	2
	Yes	36	26	Well practiced	27	28
Use the normal saline solution for cleansing surgical wound dressing.	No	0	0	Less practiced	1	0
	Yes	37	26	Well practiced	32	30
Use a face mask during dressing surgical wounds.	No	25	3	Less practiced	29	17
	Yes	12	23	Well practiced	4	13
Discard soiled materials in the proper place after performing wound dressing.	No	12	1	Less practiced	7	3
	Yes	25	25	Well practiced	26	27
Overall practice status	Good practice = 26 (41.3%)			Good practice = 30 (47.6%)		
	Poor practice = 37 (58.7%)			Poor practice = 33 (52.4%)		

### Factors associated with surgical site infection prevention practice

In bivariable analysis, the following variables: age, educational status, work experience, knowledge, attitude, having taken IP training, availability of SSIs guideline, availability of surgical supply, and continuous water supply were significantly associated with good SSIs prevention practice. Eventually, educational status, knowledge, attitude, having taken IP training, and availability of SSIs guidelines maintain their association with good SSIs prevention practice in multivariable analysis.

BSc degree nurses were 2 times more likely to practice SSIs prevention activities than diploma nurses (AOR = 2.04; 95% CI: 1.31, 3.18). Those nurses who had taken training on infection prevention were 2.23 times more likely to practice SSI prevention activities than their counterparts (AOR = 2.23; 95% CI: 1.42, 3.49). This study finding also revealed that nurses who had good knowledge and good attitudes were 1.8 and 2.6 times more likely to practice SSIs prevention activities than nurses who had poor knowledge (AOR = 1.82; 95% CI: 1.13, 2.90) and poor attitude (AOR = 2.61; 95% CI: 1.67, 4.10) respectively. Regarding SSIs prevention guidelines, nurses who were working in units having SSIs prevention guidelines were 2.45 times more likely to practice SSIs prevention activities than their counterparts (AOR = 2.45; 95% CI: 1.34, 4.47) (Table 5).

**Table 5:** Bivariable and multivariable logistic regression analysis showing the association between factors and surgical site infection prevention practice among nurses working at public hospitals in the western part of SNNPR, Ethiopia 2020, (*n* = 402).

Factors	Category	Practice status		COR (95% CI)	AOR (95% CI)
		Good	Poor		
Sex	Female	107	122	1.07 (0.72, 1.59)	0.83 (0.62, 1.02
	Male	78	95	1	
Age	≥30 years	80	60	1.99 (1.32, 3.02)	1.41 (0.83, 2.39)
	<30 years	105	157	1	1
Attitude (*n* = 402)	Good	124	78	3.62 (2.39, 5.48)	2.61 (1.67, 4.10)***
	Poor	61	139	1	1
Knowledge	Good	125	90	2.94 (1.95, 4.43)	1.82 (1.13, 2.90)*
	Poor	60	127	1	1
IP training	Trained	97	62	2.76 (1.83, 4.16)	2.23 (1.42, 3.49)***
	Not trained	88	155	1	1
Educational status	Degree	115	82	2.70 (1.80, 4.05)	2.04 (1.31, 3.18)***
	Diploma	70	135	1	1
Availability of SSIs prevention guideline	Yes	40	25	2.12 (1.23, 3.65)	2.45 (1.34, 4.47)**
	No	145	192	1	1
Work experience	≥5 years	57	40	1.97 (1.24, 3.13)	1.45 (0.78, 2.69)
	<5 years	128	177	1	1
Availability surgical supply	Yes	140	141	1.68 (1.08, 2.59)	1.14 (0.69, 1.89)
	No	45	76	1	1
Availability of continuous water supply	Yes	113	97	1.94 (1.94, 2.89)	1.40 (0.89, 2.20)
	No	72	120	1	1
Type of working hospital	Teaching	78	72	1.42 (0.81, 2.49)	1.22 (0.93, 1.51)
	General	75	103	0.96 (0.55, 1.65)	0.81 (0.45, 1.17)
	Primary	32	42	1	

COR, crude odd ratio, AOR, adjusted odd ratio, CI, confidence interval, 1, reference.

*** Significant at *p*-value less than 0.001.

** Significant at *p*-value less than 0.01.

* Significant at *p*-value less than 0.05.

## Discussion

The finding of this study revealed that the overall nurses’ good self-reported SSIs prevention practice was 46%. This finding is consistent with study findings from Bangladesh, Tanzania, Bahir-dar city, and Amhara regional state referral hospitals in Ethiopia (12, 13, 16, 17). These findings imply that the practice of nurses regarding SSIs prevention was affected by multiple factors like insufficient IP training, especially on SSIs prevention methods following the latest global and national guidelines with the latest recommendations.

The current study is not consistent with study findings from India, Nigeria, and Harar City in Ethiopia (3, 10, 15). The discrepancy in the finding from India is that it was done with subgroup analysis among a small sample, which was among only 31 nurses and 107 other health professionals. The other reason for the discrepancy is variation in the operational definition used to measure nurses’ practice. In addition to this, a study in India was done by using only a 3 Likert scale and 10 practice measuring questions. The discrepancy in findings from Nigeria is due to variations in sample size, sampling techniques, and study setting. The Nigerian study included 100 nurses who worked solely in the operating room and surgical ward of a given teaching hospital. The current study finding is higher than the study finding from Harar city. This is due to variations in their study settings. The study from Harar City was done among nurses working in all units, including units where wound care is not being performed. Therefore, the findings of the study is highly prone to recall bias and acquiescence bias. In addition to this, an event of the COVID-19 pandemic condition and its recommended international prevention activities during the current study period have an effect. For instance, SSIs prevention practices like the usage of face masks and handwashing practices were mainly affected by COVID-19 prevention practices.

BSc nurses in this study were 1.9 times more likely involved in IP training than diploma nurses. Similarly, BSc nurses in this study were also 3 times more likely to be knowledgeable and 1.8 times more likely to have a good attitude than their counterparts. This finding is in line with study findings from Addis Ababa city and Harar city in Ethiopia (15, 21). However, this finding is in contradiction with the finding from the Amhara regional state referral hospital in Ethiopia (16). This discrepancy is due to variation in the proportion of participants by their educational status. The proportion of diploma nurses in the study from Amhara regional state referral hospitals was only 8.5%. However, it was 51% in this study. Therefore, it indicates that those groups of participants who are involved in a higher proportion have a higher probability to being affected by factors affecting good SSIs prevention practice.

The current study found that nurses who had received IP training were more likely to engage in SSIs prevention activities than those who had not. This is because IP training on newly recommended national and global guidelines is one of the mechanisms to escalate nurses’ knowledge and attitude toward SSIs prevention strategies. Thus, improved nurses’ SSI prevention knowledge and attitude could improve nurses’ SSI prevention practice. For instance, those nurses who had taken IP training in this study were 2.5 and 2 times more likely to have good knowledge and a good attitude towards SSIs prevention activities than their counterparts, respectively. This finding is consistent with similar studies from Bahir-Dar City and Harar City in Ethiopia (15, 17).

The current study identified that nurses who were working in units having SSIs prevention guidelines were more likely to practice SSIs prevention activities than their counterparts. This is because if a written SSIs prevention guideline is readily available in their working units, nurses can refer to the guideline and improve their knowledge, which in turn improves their skills to perform a given task. This finding is consistent with the findings from A. A. City and southeast Ethiopia (25, 26). However, evidence from Harar City identified that the availability of SSIs prevention guidelines has no significant association with nurses’ SSIs prevention practice (15). This discrepancy is due to nurses’ variations in their utilization of available guidelines in their working units. For instance, the reported utilization of available guidelines by nurses in this study was 93.3%.

In addition, this study's findings confirmed that nurses who had good knowledge were more likely to practice SSIs prevention activities than those who had poor knowledge. This is due to the fact that knowledge is a critical component of nursing decision-making and an important element in forming the right attitude. For instance, those nurses who had good knowledge about SSIs prevention practice in this study were four times more likely to have a good attitude than those who had poor knowledge. Therefore, nursing practice regarding SSIs prevention cannot be accomplished without nursing SSIs prevention knowledge and the right attitude towards that acquired knowledge. This finding is consistent with the findings across all related pieces of literature; like from Nigeria and Harar City in Ethiopia (14, 15).

Similarly, the current study also affirmed that nurses who had a good attitude toward SSI prevention activities were more likely to practice SSIs prevention activities than those who had a poor attitude. This is due to the reason that attitude is much more likely to affect behavior, so nurses who had a good attitude were more likely to engage in SSIs prevention activities. Although knowledge is important in shaping the right attitude, it is not certain that the knowledge gained will translate into practice. Therefore, a good attitude toward SSIs prevention strategies is the key element to translating acquired knowledge into practice. This finding is also consistent with the findings of all related pieces of literatures; like those from Nigeria and Harar City in Ethiopia (14, 15).

### Strength of the study

This study had established some important points which will help us to generate a hypothesis. It had shown the surgical site infection prevention practice and used it to show the relationship between Surgical Site Infection Prevention Practice and factors.

### Limitation of the study

Since the study was cross-sectional, it couldn't establish the cause and effect relationships.

## Conclusion

Surgical site infection prevention practice of nurses who were working in selected units at the public hospital in the western part of SNNPR was low, which is significantly associated with being BSc degree, having taken IP training, and working in the units where SSIs prevention guideline available, having good knowledge and having a good attitude. So, nurses in this study area should upgrade their knowledge by referring to SSI prevention-related guidelines/manuals if available in their working unit and by reading the new evidence-based practices regarding SSI prevention activities. The hospitals’ management bodies and infection prevention committees should emphasize the importance of adherence to newly recommended SSIs prevention guidelines by developing their guideline based on new evidence-based WHO recommendations regarding SSIs prevention which is strongly recommended.

## Data Availability

The raw data supporting the conclusions of this article will be made available by the authors, without undue reservation.
